# Prevalence of cardiovascular disease in type 2 diabetes: a systematic literature review of scientific evidence from across the world in 2007–2017

**DOI:** 10.1186/s12933-018-0728-6

**Published:** 2018-06-08

**Authors:** Thomas R. Einarson, Annabel Acs, Craig Ludwig, Ulrik H. Panton

**Affiliations:** 1 Leslie Dan Faculty of Pharmacy, University of Toronto, Barrie, Canada; 2 Last Mile, Holte, Denmark; 3 Novo Nordisk A/S, Søborg, Denmark

**Keywords:** Cardiovascular disease, Type 2 diabetes, Prevalence, Stroke, Ischemic heart disease, Myocardial infarction, Angina

## Abstract

**Background:**

Cardiovascular disease (CVD) is a common comorbidity in type 2 diabetes (T2DM). CVD’s prevalence has been growing over time.

**Purpose:**

To estimate the current prevalence of CVD among adults with T2DM by reviewing literature published within the last 10 years (2007–March 2017).

**Methods:**

We searched Medline, Embase, and proceedings of major scientific meetings for original research documenting the prevalence of CVD in T2DM. CVD included stroke, myocardial infarction, angina pectoris, heart failure, ischemic heart disease, cardiovascular disease, coronary heart disease, atherosclerosis, and cardiovascular death. No restrictions were placed on country of origin or publication language. Two reviewers independently searched for articles and extracted data, adjudicating results through consensus. Data were summarized descriptively. Risk of bias was examined by applying the STROBE checklist.

**Results:**

We analyzed data from 57 articles with 4,549,481 persons having T2DM. Europe produced the most articles (46%), followed by the Western Pacific/China (21%), and North America (13%). Overall in 4,549,481 persons with T2DM, 52.0% were male, 47.0% were obese, aged 63.6 ± 6.9 years old, with T2DM duration of 10.4 ± 3.7 years. CVD affected 32.2% overall (53 studies, N = 4,289,140); 29.1% had atherosclerosis (4 studies, N = 1153), 21.2% had coronary heart disease (42 articles, N = 3,833,200), 14.9% heart failure (14 studies, N = 601,154), 14.6% angina (4 studies, N = 354,743), 10.0% myocardial infarction (13 studies, N = 3,518,833) and 7.6% stroke (39 studies, N = 3,901,505). CVD was the cause of death in 9.9% of T2DM patients (representing 50.3% of all deaths). Risk of bias was low; 80 ± 12% of STROBE checklist items were adequately addressed.

**Conclusions:**

Globally, overall CVD affects approximately 32.2% of all persons with T2DM. CVD is a major cause of mortality among people with T2DM, accounting for approximately half of all deaths over the study period. Coronary artery disease and stroke were the major contributors.

## Background

The International Diabetes Federation (IDF) estimates that worldwide, 415 million people have diabetes, 91% of whom have type 2 diabetes mellitus (T2DM) [1]. People with diabetes comprise 8.8% of the world’s population, and IDF predicts that the number of cases of diabetes will rise to 642 million by 2040 [1]. The prevalence of T2DM has been steadily increasing over time. Using data from the Framingham Heart Study, Abraham et al. [2] noted that the overall annualized incidence rates of the disease per 1000 persons increased from 3.0 in the 1970s to 5.5 in the first decade of the 2000s. That change represented an increase in the incidence of T2DM of 83.3% and was higher in males than females by a factor of 1.61.

Cardiovascular disease (CVD) is a major cause of death and disability among people with diabetes [1, 3]. Adults with diabetes historically have a higher prevalence rate of CVD than adults without diabetes [4], and the risk of CVD increases continuously with rising fasting plasma glucose levels, even before reaching levels sufficient for a diabetes diagnosis [5].

T2DM reduces life expectancy by as much as 10 years, and the main cause of death for patients with T2DM is CVD [1, 3]. Furthermore, people with T2DM are disproportionately affected by CVD compared with non-diabetic subjects [6]. Haffner et al. [6] reported death rates due to cardiovascular causes over a 7-year period in patients with and without T2DM. In persons with T2DM, the death rates were 15.4% for those with no prior myocardial infarction (MI) and 42.0% in patients having a history of MI. In contrast, patients who did not have T2DM, the death rates due to cardiovascular causes were 2.1 and 15.9%, respectively.

In the Framingham Heart Study, Fox [7] reported that, along with the increasing T2DM prevalence, the attributable risk of CVD due to T2DM increased from 5.4% in the period 1952–1974 to 8.7% in the period 1975 and 1998. In a longitudinal study of 881 patients with T2DM over 10 years, van Hateren et al. [8] indicated that the hazard ratio for death due to CVD was constantly increasing each year. Thus, an increasing burden of diabetes will likely be followed by an increasing burden of CVD.

Given the clinical burden that CVD complications have on T2DM patients, there has been an increased focus on the joint management of T2DM and CVD. Good glycemic control remains the main foundation for managing T2DM. Although the importance of intensive glycemic control for protection against microvascular complications and CVD in people with T1DM is well established [9, 10], its role for reducing cardiovascular risk has not been established as clearly in people with T2DM [11–13]. Hence, the most effective approach for prevention of macrovascular complications appears to be multifactorial risk factor reduction (glycemic control, smoking cessation, diet, exercise, aggressive blood pressure control, treatment of dyslipidemia).

As a result, diabetes treatment guidelines have been updated to provide guidance on how to prevent and manage the onset of CVD [14]. Furthermore, there is increasing pressure from regulatory agencies that antidiabetic treatments demonstrate cardiovascular safety and benefits, especially for major cardiovascular events such as cardiovascular mortality, non-fatal MI, and stroke [15, 16]. Following these regulatory requirements, several cardiovascular outcomes trials (CVOT) have been completed, which demonstrate that certain anti-diabetic treatments are associated with a lower risk of CVD [17–20].

The increased focus on adequately treating patients with both CVD and T2DM requires that we have updated prevalence rates of CVD among patients with T2DM. This is especially needed to inform clinical and policy level decision-making by healthcare providers, healthcare policy decision-makers, and health economic analysts. Reviews have been published on the epidemiology of type 1 diabetes (T1DM), and CVD [21], pre-diabetes and the risk of CVD [22], or reviews have focused on specific countries [23]. However, there is no recent global review on the prevalence of CVD among adults with T2DM. Therefore, the objective of this systematic literature review was to quantitatively summarize rates of prevalence of CVD in adults with T2DM in studies published during the past 10 years.

Although CVD is an umbrella term that includes coronary artery disease (CAD), cerebrovascular disease (CBV), and peripheral vascular disease, the focus of this review was on CVD outcomes that are relevant to major cardiovascular events. Therefore, the review specifically focused on the prevalence of CAD and CBV. CAD has many synonyms, including ischemic heart disease, coronary heart disease (CHD), atherosclerotic heart disease, and atherosclerotic CVD. Conditions within this category are stable angina pectoris, unstable angina pectoris, MI (also known as heart attack), and sudden cardiac death (SCD). CBV comprises mainly stroke (intracerebral hemorrhage, cerebral infarction, cerebral arterial disease), but also may include transient ischemic attacks.

## Methods

This review was undertaken in adherence to the PRISMA Statement for systematic reviews [24].

### Eligibility criteria

Criteria for eligibility were guided by the PICO reporting system (which describes the participants, interventions, comparisons, and outcome[s] of the systematic review), together with the specification of the type of study design (PICOS), from the Preferred Reporting Items for Systematic Reviews and Meta-Analyses (PRISMA) [24].

#### Participants

Included in this research were adult patients ≥ 18 years old who had been diagnosed with T2DM.

#### Interventions

Not applicable in this research.

#### Comparisons

Prevalence rates of CVD between males and females, and between obese and non-obese patients were compared. It was acknowledged that, in the literature, authors often used different terms or combinations of terms to describe their patients. The aim was to be all-inclusive in order to capture all relevant patient populations. Broad definitions of acceptable diseases were CVD, CAD, CHD, ischemic heart disease (IHD), congestive heart failure (CHF), or CBV. Specific conditions of interest included stroke, MI/heart attack, angina pectoris, heart failure, and atherosclerosis as well as cardiovascular or cardiac death.

Excluded were other forms of CVD including peripheral artery disease (PAD), rheumatic heart disease, cardiac dysrhythmias (e.g., atrial or ventricular fibrillation), or requirement for surgery such as coronary artery bypass grafting (CABG)/coronary revascularization. Also excluded were intermediate states such as hypertension or metabolic syndrome or studies of carotid intima-media thickness (CIMT).

#### Outcome[s]

The outcome of interest was the prevalence of each of these diseases/outcomes, then aggregated by continent/IDF Region, by country, and by the country’s economic status.

#### Study design

The primary focus was on prevalence studies and cross-sectional surveys, including database studies or patient chart reviews. Incidence studies were accepted only if they provided population-based baseline and follow-up data. Included were peer-reviewed studies published in any language. Both published articles and abstracts from scientific meetings were eligible. However, any published studies from clinical trial programs or individual pharmaceutical products were excluded.

### Information sources and search strategy

The search was undertaken between February 15 and March 6, 2017. Databases searched included Medline and Embase between January 2007 and March 2017. In addition, PubMed was searched from 2014 to identify articles that were “ahead of print” yet fully available. Evidence presented at selected conferences during the last 5 years were accessed, including the Annual Meetings of the International Society Pharmacoeconomic Outcomes and Research (ISPOR), American Diabetes Association (ADA), European Association for the Study of Diabetes (EASD) and American Association of Clinical Endocrinologists (AACE). Keywords linked to MeSH terms specific to each database were used in the search including prevalence, OR epidemiology, AND acute coronary syndrome, OR cardiovascular disease, OR cardiovascular death, OR non-fatal myocardial infarction, OR non-fatal stroke, OR obesity AND type 2 diabetes mellitus. Other keywords were cerebrovascular disease, cerebral arterial disease, intracerebral hemorrhage, cerebral infarction, coronary artery disease, ischemic heart disease, atherosclerotic heart disease, coronary heart disease, angina pectoris. Identified articles and previous reviews were hand searched for articles that may have included data useful to this search.

### Article identification and selection

Two reviewers independently searched Medline, Embase and the proceedings of major scientific meetings for suitable papers. Results were compared and adjudicated through consensus discussion. A third reviewer checked all results for quality assurance.

### Data collection

Data extracted from articles included information concerning the publication, the patients involved, and outcomes of interest. Publication items included the first author, year of publication, the country in which the data were collected, and date of data collection. Patient data collected included the number of patients screened, percentages of males and females, average age, duration of T2DM, the proportion with obesity (or average body mass index (BMI) ± SD). Outcome data consisted of the numbers and percentages of patients having each cardiovascular outcome, overall and separately for males and females, where available. The same procedure (two independent reviewers plus a third judge) was followed for data collection as for article selection.

### Data analysis

Data were analyzed descriptively, with sums, averages, and medians, and ranges reported. The primary outcome was the estimate of prevalence rates of CVD in patients with T2DM. No overall quantitative synthesis was undertaken. Weighted averages were calculated for individual countries and IDF regions. For patient characteristics, we calculated simple averages and medians across studies. Due to a single study with a sample size of more than three million people, which skewed the data, we calculated weighted averages for patient characteristics with and without that study. It should be noted that averages were based on the studies that reported the outcome, which may then represent a subgroup of the entire pool of studies.

The risk of bias was explored by applying the checklist from the STROBE initiative [25]. They have produced a validated checklist of items that should be addressed in reports of observational studies. There are 22 main items, each of which addresses an issue of research design and is presented in a list of recommendations. Items are scored as dichotomously as acceptable or not acceptable.

## Results

### Included studies

**Fig. 1:**
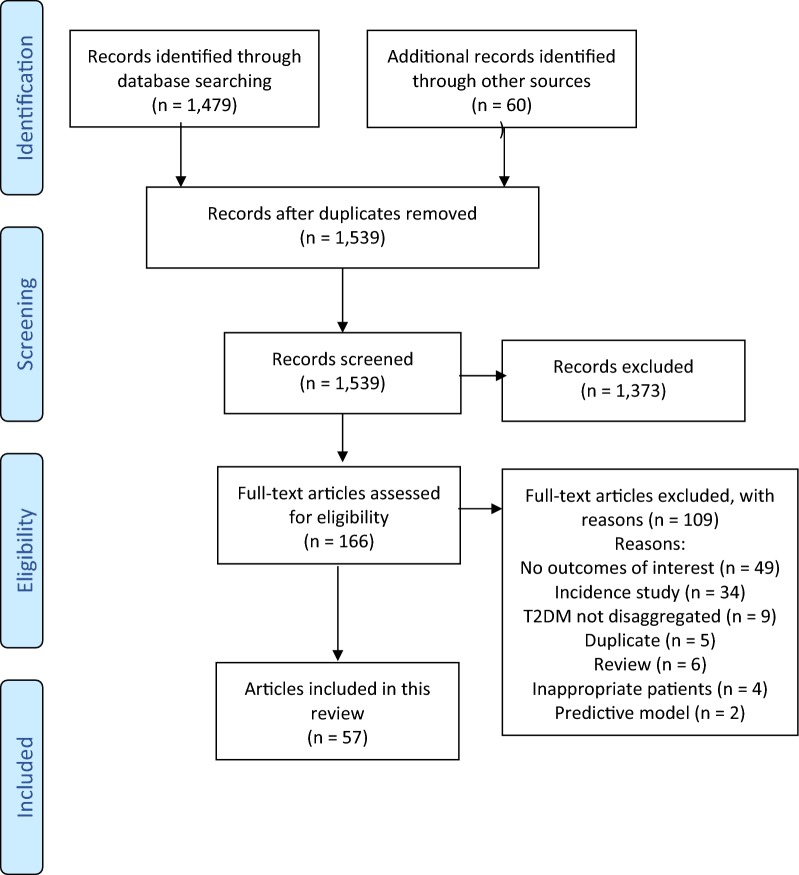
Flowchart of article selection. The flowchart depicts the article selection process

The flowchart in Fig. 1 depicts the article selection process. We initially identified 1539 papers that appeared to be suitable, but after examining them systematically, 57 studies were accepted. Three articles each presented two different sets of results [26–28]; therefore, there are 60 sets of analyses within these 57 articles. Table 1 lists these studies along with their descriptive variables. There were 51 full articles and six abstracts presented at scientific meetings. Data collectively represent more than 4.5 million people with T2DM from around the globe.

**Table 1:** Overview of studies in the analysis

Author (year)	Country	Patients	% obese or reported BMI	% males	Age (years)	Diabetes duration (years)	Follow-up (years)	Time of data collection
Alaboud (2016) [41]	Saudi Arabia	748	64.3%	42.4%	57.9	13.3	NR	Apr–Jun 2014
Alonso-Moran (2014) [42]	Spain	134,421	NR	54.0%	NR	NR	NR	2007–2011
Alwakeel (2008) [30]	Saudi Arabia	1952	44.8%	48.3%	58.4	10.4	7.9	Jan 1989–Jan 2004
Bhatti (2016) [26]^a^	India	1522	BMI = 26.7 ± 4.4	58.3%	58.1	7.2	NR	2011–2014
Boonman-de Winter (2012) [38]	Netherlands	581	28.1%	53.4%	71.6	5.5	NR	Feb 2009–Mar 2010
Cardoso (2008) [31]	Brazil	471	NR	34.2%	60.5	9.3	4.8	1994–1996, 2001
Carnethon (2010) [32]	USA	919	BMI = 28.2 ± 4.9	53.4%	72.8	NR	11.3	(1989/92–93) through 2005
Carrasco-Sánchez (2014) [43]	Spain	490	BMI = 31.4 ± 14.23	44.3%	76.6	NR	NR	2008–2011
Cheng (2014) [86]	China	2834	91.6%	51.8%	58.5	7.0	NR	Aug 2011–Mar 2012
Collier (2015) [27]^a^	Scotland	7385	51.0%	NR	64.3	NR	NR	NR
		6032	57.5%	NR	66.4	NR	NR	NR
Cortez-Dias (2010) [87]	Portugal	3215	45.1%	38.4%	58.1	NR	NR	Apr 2006–Nov 2007
Daghash (2007) [88]	Qatar	180	BMI = 30.35 ± 4.9	43.0%	51.3	NR	NR	May–Oct 2004
Doucet (2016) [89]	France	987	BMI = 29.7 ± 5.2	47.9%	77.1	17.8	NR	Jun 2009–Jul 2010
Eeg-Olofsson (2010) [33]	Sweden	18,334	BMI = 28.8 ± 5	56.7%	64.0	8.0	5.6	1997–1998–2003
Farrell (2014) [90]	Ireland	309	NR	NR	NR	NR	NR	NR
Fu (2010) [91]	Spain, France, UK, Norway, Finland, Germany, Poland	1942	52.9%	64.4%	64.5	6.2	2.8	Jun 2006–Feb 2007
Giallauria (2015) [92]	Italy	475	NR	74%	69.7	NR	NR	Jan 28–Feb 10, 2008
Glogner (2014) [40]	Sweden	83,021	BMI = 28.9 ± 5.04	55.3%	65.8	7.6	7.2	Enrolled: 1998–2003; through 2009
Gobardhan (2017) [93]	Netherlands	318	53.0%	50.9%	52.3	11.0	10.0	NR
Gondim (2016) [94]	Brazil	66	BMI = 27.17 ± 4.62	43.9%	64.6	NR	NR	NR
Hermans (2016) [95]	Belgium	711	BMI = 29.5 ± 5.8	66%	67.0	16.0	NR	NR
Hunt (2014) [96]	USA	1030	BMI = 33.6	23.5%	52.7	10.5	NR	1995–2003
Jackson (2012) [97]	Scotland	216,652	NR	53.6%	≥40	NR	4.5	2001–2007
Jurado (2009) [98]	Spain	307	44.9%	61.6%	59.6	8.5	NR	Nov 2001–Dec 2002
Kucharska-Newton (2010) [99]	USA	209	BMI = 31.0 ± 6.0	43.5%	55.5	NR	NR	1987–1989–2001
Kwon (2014) [100]	Korea	59	NR	59.3%	64.5	NR	13.0	Korea
Lin (2013) [45]	USA	162,332	NR	NR	≥18	≥2	≥2	USA
Liu (2015) [101]	China	21,072	NR	53.9%	63.7	NR	NR	China
Luo (2014) [102]	China	4836	BMI: 24.3	57.6%	64.9	NR	1.0	China
MacDonald (2011) [103]	247 countries	669	BMI = 31.2 ± 4.6	51.7%	58.8	7.2	2.0	247 countries
Malik (2015) [34]	Scotland	121,523	BMI = 31.7 ± 6.6	52.0%	63.0	4.2	4.8	Scotland
Mansour (2013) [48]	Iraq	1079	33.8%	58.8%	56.3	7.4	NR	Iraq
Mazza (2007) [104]	Italy	581	30.1%	34.8%	74.3	20.3	12.0	Italy
Menghua (2014) [49]	China	240	NR	NR	NR	NR	NR	China
Menzaghi (2014) [36]	Italy	2094	BMI = 29.1 ± 5.3	51.3%	61.9	10.4	12.9	Italy
Mody (2007) [105]	USA	4816	NR	34.4%	50.9	NR	NR	USA
Mundet (2012) [106]	Spain	4298	BMI = 29.34 ± 4.84	48.2%	67.4	8.4	10.0	Spain
Narksawat (2013) [107]	Thailand	1505	32.2%	30.4%	63.3	NR	NR	Thailand
Norhammar (2016) [50]	Sweden	352,436	NR	56.1%	67.1	NR	NR	Sweden
Penno (2015) [108]	Italy	11,538	34.5%	52.9%	65.5	12.5	NR	Italy
Rodriguez-Poncelas (2014) [109]	Spain	1141	46.5%	60.6%	66.8	9.1	NR	Feb–Jul 2011
Rossi (2011) [51]	Italy	5181	BMI = 29.8 ± 5.0	58.4%	64.4	10.0	2.3	(Jan 2006–Nov 2007), 2009
Salinero-Fort (2016) [35]	Spain	3407	BMI = 30.1 ± 4.9	50.3%	69.0	9.1	5.0	2007 (2008–2012)
Senthil (2014) [69]	India	134	NR	72.1%	NR	NR	NR	NR
Shestakova (2016) [29]	Russian Federation	3,060,517	NR	28.3%	NR	NR	1.0	2014–2015
Soetedjo (2014) [110]	Indonesia	400	56.8%	43.8%	57.7	10.3	NR	Dec 2013–Jun 2014
Song (2009) [46]	UK	2733	BMI = 33.4 ± 6.7	NR	64.2	12.7	NR	2008
Suh (2008) [111]	USA	608	51.4%	44.82%	73.2	12.9	10.0	1999–2004
Tamba (2013) [37]	Cameroon	132	30.0%	56%	58.0	12.0	6.0	2000–2009
Tan (2016) [28]^a^	Australia	793	54.8%	50.9%	67.2	8.0	15.0	2008–2011
		65	35.4%	56.9%	61.1	10.0	15.0	
Utrera-Lagunas (2013) [112]	Mexico	160	33.8%	45.0%	69.2	18.3	NR	Feb 2011–Jan 2012
Vinagre (2012) [113]	Spain	286,791	45.4%	53.7%	68.2	6.5	NR	2009
Wentworth (2012) [39]	Australia	711	>50%	55.1%	53.0	11.4	NR	1998–2011
Wong (2012) [114]	USA	889	NR	46.2%	60.6	13.3	NR	2003–2006
Yan (2015) [115]	Hong Kong	10,952	63.6%	56.1%	58.2	7.0	NR	Nov 2007–Jul 2012
Yang (2015) [47]	Korea	595	BMI = 24.29 ± 3.15	58.32%	64.9	13.6	NR	2006–2010
Zekry (2012) [116]	Switzerland	83	BMI = 27.2 ± 4.9	36.1%	84.2	NR	4.0	Jan 2004–Dec 2005
57 studies	Total	4549,481						
	Median	1030	45.4%	52.0%	64.3	10.0	6.0	
	Average	77,110	47.0%	50.5%	63.6	10.4	7.3	
	SD (range)	(59–3,060,517)	14.7%	10.3%	6.9	3.7	4.5	

*BMI* body mass index, *NR* not reported, *SD* standard deviation

^a^Study reports two separate analyses within the same paper; thus, there are 60 studies in 57 articles

In Table 2, results are presented geographically according to the classification system used by the IDF [1]. Studies from 25 countries were represented in this review: Australia, Belgium, Brazil, Cameroon, China, France, India, Indonesia, Iraq, Ireland, Italy, Korea, Mexico, Netherlands, Portugal, Qatar, Russian Federation, Saudi Arabia, Scotland, Spain, Sweden, Switzerland, Thailand, UK and USA. Details are provided in Table 3. Three areas were responsible for generating 80% of the studies. Europe produced most articles (46%), followed by the Western Pacific/China (21%), and North America (13%). The other 20% were from the rest of the world. There were no discernible patterns differentiating prevalence rates between countries, based on income status. Part of the problem is that there are few studies in low- and middle-income countries and none from those in the lowest income level.

**Table 2:** Geographic distribution of prevalence studies of cardiovascular disease in type 2 diabetes mellitus

Region	Population^a^ (millions)	Studies	N	Stroke (%)	MI	Angina	Heart failure	Atherosclerosis	CAD	CVD (%)
Africa	441	1	132	5.0	NR	NR	NR	NR	23.6%	28.6
Europe	660	29^b^	4,327,503	7.2	10.0%	14.6%	19.0%	33.0%	15.4%	30.0
Middle East and North Africa	387	4	3959	7.1	11.4%	NR	NR	NR	27.4%	26.9
North America and Caribbean	344	8	170,963	10.9	13.6%	17.2%	29.5%	NR	20.1%	46.0
South and Central America	315	2	537	5.5	NR	NR	4.2%	NR	22.6%	27.5
Southeast Asia	926	3^b^	1656	3.1	NR	NR	NR	NR	29.4%	42.5
Western Pacific (includes China)	1600	12^b^	44,062	11.4	NR	NR	4.3%	26.0%	23.6%	33.6
Multiple countries	NR	1	669	1.9	3.9%	9.9%	0.7%	NR	NR	16.4
Total^c^	4673	60	4,549,481	7.6	10.0%	14.6%	14.9%	29.1%	21.2%	32.2

*CAD* coronary artery disease, *CVD* cardiovascular disease (includes all complications), *MI* myocardial infarction, *NR* not reported

^a^Adults aged 20–79. Source: IDF Atlas 2015 [1]

^b^A study reports two separate analyses within the same paper; thus, there are 60 studies in 57 articles

^c^Prevalence rates weighted by inverse variance

**Table 3:** Number of studies and cardiovascular outcomes reported, by country

Country	Income status^a^	Studies	Patients	Stroke	MI	Angina	CHF	Atherosclerosis	CAD	CVD (%)
Australia	High	3^b^	1569	7.7%	NR	NR	NR	NR	21.8%	29.4
Belgium	High	1	711	8.0%	NR	NR	NR	NR	22.0%	30.0
Brazil	Upper middle	2	537	5.5%	NR	NR	4.2%	NR	22.6%	27.5
Cameroon	Low middle	1	132	5.0%	NR	NR	NR	NR	23.6%	28.6
China	Upper middle	5	39,934	15.2%	NR	NR	4.3%	30.5%	17.0%	28.4
France	High	1	987	15.3%	NR	NR	9.1%	NR	29.5%	53.9
India	Low middle	3^b^	1656	3.1%	NR	NR	NR	NR	29.4%	42.6
Indonesia	Low middle	1	400	10.8%	NR	NR	NR	NR	28.8%	39.6
Iraq	Upper middle	1	1079	NR	NR	NR	NR	NR	NR	16.0
Ireland	High	1	309	5.2%	NR	NR	NR	NR	17.8%	23.0
Italy	High	5	19,869	2.4%	6.2%	NR	NR	NR	11.1%	14.8
Korea	High	2	654	20.3%	NR	NR	NR	21.5%	37.0%	47.2
Mexico	Upper middle	1	160	NR	NR	NR	57.5%	NR	NR	57.5
Netherlands	High	3	899	6.1%	NR	NR	30.6%	33.0%	28.2%	44.5
Portugal	High	1	3215	5.0%	NR	NR	NR	NR	12.0%	17.0
Qatar	High	1	180	NR	NR	NR	NR	NR	31.7%	31.7
Russian Federation	Upper middle	1	3,060,517	4.2%	3.4%	NR	NR	NR	13.4%	21.0
Saudi Arabia	High	2	2700	7.1%	11.4%	NR	NR	NR	23.1%	30.0
Scotland	High	3^b^	351,592	NR	NR	NR	NR	NR	19.7%	19.7
Spain	High	7	430,855	8.0%	20.2%	NR	30.3%	NR	13.6%	29.8
Sweden	High	3	453,791	10.3%	9.3%	14.6%	9.5%	NR	NR	31.3
Switzerland	High	1	83	15.7%	NR	NR	NR	NR	33.7%	49.4
Thailand	Upper middle	1	1505	2.5%	NR	NR	NR	NR	NR	2.5
UK	High	1	2733	7.9%	NR	NR	NR	NR	34.5%	42.4
USA	High	7	170,803	10.9%	13.6%	17.2%	15.5%	NR	28.1%	44.0
16 countries	High	42	1,440,950	9.3%	12.1%	15.9%	19.0%	27.3%	24.3%	33.6
6 countries	Upper middle	11	3,103,732	6.9%	3.4%		22.0%	30.5%	17.7%	25.5
3 countries	Low middle	5	2188	6.3%					27.3%	36.9
0 countries	Low	0	0							
25 countries	Overall	58	4,546,870^c^	8.4%	10.7%	15.9%	20.1%	28.3%	23.7%	32.1

*CAD* coronary artery disease (also reported as coronary heart disease or ischemic heart disease), *CHF* congestive heart failure, *CVD* cardiovascular disease, *MI* myocardial infarction, *NR* not reported

^a^World Bank status [117] based on Gross National Income per capita per annum (in USD) as of 01 July, 2016: Low < $1025; Low middle = $1026–$4035; Upper middle = $4036–$12,475; High > $12,476. Country status extracted from World Bank Database [118]

^b^A study reports two separate analyses within the same paper. In total there are 58 studies across 55 articles

^c^Study evaluated patients from multiple countries and therefore is not included in this table. Thus, there are 4,546,870 patients across 55 studies

### Patient characteristics

In the 57 individual studies, data from 4.5 million people with T2DM were presented with nearly 3.1 million people coming from a single Russian study by Shestakova [29]. As presented in Table 1, using a simple average across studies, the average age was 63.6 ± 6.9 (median = 64.3 years; weighted average = 66.3 ± 6.9 years). The weighted average proportion of persons with obesity was 46.3 ± 15.0%, with a simple average of 47.0 ± 14.7% (median = 45.4%), defined as a BMI ≥ 30 kg/m^2^. The mean percentage of males across the studies was 50.5 ± 10.3% (median = 52.0%); the weighted average of the proportion of males was 36.0 ± 10.0%, including the study by Shestakova [29], and 54.1 ± 9.9% excluding that study. The patients had T2DM for an average duration of 10.4 ± 3.7 years (median = 10.0 years; weighted average = 6.6 ± 3.7 years). Among the 23 studies that reported duration of follow-up, the average was 7.3% ± 4.5 years (median = 6.0 years; weighted average = 5.2 ± 4.3 years).

Prevalence rates of cardiovascular comorbidities are summarized in Table 4 for all patients as well as separately for males and females. In studies reporting gender-specific prevalence rates, males had higher prevalence rates than females for all outcomes except overall CVD, where both sexes had an overall prevalence rate of approximately 27%. Overall, in studies that presented prevalence rates for males and females combined, the prevalence of CVD among persons with T2DM was 32.2%. CAD and atherosclerosis were the most prevalent CVD comorbidities, with prevalence rates of 21.2 and 29.1%, respectively, whereas stroke was the least prevalent with a prevalence rate of 7.6%. It is unclear why people with T2DM have different susceptibilities to these diseases. An explanation for the high prevalence rate for atherosclerosis could be that it is an artifact of patient selection. In the studies that examined atherosclerosis, most patients were enrolled if they had high-risk scores for atherosclerosis, resulting in a very high rate of disease detection.

**Table 4:** Summary of prevalence rates of cardiovascular comorbidities in persons with type 2 diabetes

Sex	Cardiovascular outcome	Studies	N	Rate^a^ (%)	95% confidence interval (%)
Both	Stroke	39	3,901,505	7.6	6.6–8.6
	Myocardial infarction	13	3,518,833	10.0	7.5–12.5
	Angina pectoris	4	354,743	14.6	12.0–17.3
	Heart failure	14	601,154	14.9	13.0–16.7
	Atherosclerosis	4	1153	29.1	21.7–36.4
	Coronary artery disease	42	3,833,200	21.2	20.3–22.2
	Cardiovascular disease (any)	53	4,289,140	32.2	30.0–34.4
Males^b^	Stroke	10	232,525	6.7	6.0–7.3
	Myocardial infarction	2	1170	11.9	4.3–19.5
	Angina pectoris	1	454	21.1	16.3–26.9
	Heart failure	4	73,361	25.3	11.4–39.2
	Coronary artery disease	9	237,367	18.7	16.5–20.8
	Cardiovascular disease	16	241,406	27.6	25.3–29.9
Females^b^	Stroke	10	202,348	5.9	5.1–6.7
	Myocardial infarction	2	1812	9.8	3.5–16.0
	Angina pectoris	1	803	17.4	15.0–20.2
	Heart failure	4	62,690	24.0	11.2–36.8
	Coronary artery disease	10	205,493	14.3	12.4–16.1
	Cardiovascular disease	16	209,153	27.2	22.7–31.7

^a^Weighted by inverse variance

^b^No studies reported atherosclerosis for males or females; only in the aggregate. Rates for males and females do not sum to the total as not all studies reported all outcomes

### CVD mortality among patients with T2DM

Table 5 presents the data regarding the rates of mortality associated with CVD in persons with T2DM. The weighted average of death rates from the eight studies with 3,208,557 patients with T2DM was 9.9% (95% CI 8.6–11.3%) [29–36]. There were 6.3% who died due to CAD and another 1.5% from CBV. Comparing patients with both T2DM and CVD with patients having neither T2DM nor CVD, the odds ratio for death was 4.56 (95% CI 3.53–5.89) [32]. Using a weighted average from seven studies (N = 86,557) [29–35], the calculated deaths due to CVD comprised 50.3% (95% CI 37.0–63.7%) of all deaths in patients with T2DM. The major contributors were CAD, which was responsible for 29.7% (95% CI 25.1–34.4%) and stroke/CBV for 11.0% (95% CI 8.8–13.3%).

**Table 5:** Mortality associated with cardiovascular disease in persons with type 2 diabetes

Disease	Author (year)	Data collection period	Patients	n	All deaths	%	CVD Deaths	% Death rate	% CVD proportion of all deaths
CVD	Alwalkeel (2008) [30]	Jan 1989–Jan 2004	T2DM adults	1952	161	8.20%	97	5.00%	60.20%
CVD	Cardoso (2008) [31]	1994–96 to 2001	T2DM adults	471	121	25.70%	44	9.30%	36.40%
CVD	Carnethon (2010) [32]	1989–93 to 2005	T2DM only	659	468	71.00%	211	32.0%	45.10%
CVD	Carnethon (2010) [32]	1989–93 to 2005	CVD only	868	620	71.40%	304	35.0%	49.00%
CVD	Carnethon (2010) [32]	1989–93 to 2005	T2DM + CVD	260	219	84.20%	129	49.60%	58.90%
CVD	Carnethon (2010) [32]	1989–93 to 2005	No T2DM or CVD	3997	2095	52.40%	710	17.8%	33.90%
CVD	Eeg-Olofsson (2010) [33]	1997–98 to 2003	T2DM adults	18,334	1902	10.40%	1456	7.90%	76.60%
CVD	Malik (2015) [34]	2005–2011	T2DM adults	121,523	17,637	14.50%	3722	3.10%	21.10%
CVD	Menzaghi (2014) [36]	2001–2008	GHS study—males	242	NR	–	42	17.40%	–
CVD	Menzaghi (2014) [36]	2001–2008	GHS study—females	117	NR	–	16	13.70%	–
CVD	Menzaghi (2014) [36]	1993–1999	HPFS study—males	833	NR	–	146	17.50%	–
CVD	Menzaghi (2014) [36]	1976–1990	NMS study—females	902	NR	–	144	16.00%	–
CVD	Salinero-Fort (2016) [35]	2007–08 to 2012	T2DM adults	2442	203	8.30%	96	3.90%	47.30%
CVD	Salinero-Fort (2016) [35]	2007–08 to 2012	T2DM adults + kidney disease	965	221	22.90%	124	12.80%	56.10%
CVD	Shestakova (2016) [29]	2015	T2DM adults	3,060,516	66,093	2.20%	30,560	1.00%	46.20%
	All CVD death		Patients with T2DM	3,208,557	86,557^a^	42.3%^a^	36,576^a^	9.9%^a^	50.3%^a^
CAD	Cardoso (2008) [31]	1994–1996 to 2001	T2DM adults	471	121	25.70%	30	6.40%	24.80%
CAD	Carnethon (2010) [32]	1989–1993 to 2005	T2DM only	659	468	71.00%	132	20.00%	28.20%
CAD	Carnethon (2010) [32]	1989–1993 to 2005	CVD only	868	620	71.40%	213	24.50%	34.40%
CAD	Carnethon (2010) [32]	1989–1993 to 2005	T2DM + CVD	260	219	84.20%	111	42.70%	50.70%
CAD	Carnethon (2010) [32]	1989–1993 to 2005	No T2DM or CVD	3997	2095	52.40%	425	10.60%	20.30%
CAD	Jackson (2012) [97]	2001–2007	Male diabetics	116,145	22,033	19.00%	6000	5.20%	27.20%
CAD	Jackson (2012) [97]	2001–2007	Male non-diabetics	2,433,748			36,801	1.50%	
CAD	Jackson (2012) [97]	2001–2007	Female diabetics	100,507	20,571	20.50%	4554	4.50%	22.10%
CAD	Jackson (2012) [97]	2001–2007	Female non-diabetics	2,630,482			32,449	1.20%	
	All CAD death		Patients with T2DM	218,462^a^	42,944^a^	24.9%^a^	10,695^a^	6.3%^a^	29.7%^a^
CHF	Mazza (2007) [104]	1983–1985 to 1997	Male diabetics	202	–	–	22	10.90%	–
CHF	Mazza (2007) [104]	1983–1985 to 1997	Female diabetics	379	–	–	29	7.70%	–
CHF	Mazza (2007) [104]	1983–1985 to 1997	All	581	369	63.50%	–	–	13.80%
CHF	Shestakova (2016) [29]	2015	T2DM adults	3,060,516	66,093	2.20%	18,963	0.60%	28.70%
	All CHF deaths		Patients with T2DM	3,061,097^a^	66,093^a^	2.2%^a^	1,9104^a^	6.1%^a^	28.7%^a^
MI	Shestakova (2016) [29]	2015	T2DM adults	3,060,516	66,093	2.20%	3393	0.10%	5.10%
SCD	Kucharska-Newton (2010) [99]	1987–89 to 2001	T2DM adults	1550	NR	–	69	4.50%	–
SCD	Kucharska-Newton (2010) [99]	1987–89 to 2001	No T2DM	12,428	NR	–	140	1.10%	–
Stroke	Jackson (2012) [97]	2001–2007	Male diabetics	116,145	22,033	19.00%	1942	1.70%	8.80%
Stroke	Jackson (2012) [97]	2001–2007	Male non-diabetics	2,433,748			13,191	0.50%	
Stroke	Jackson (2012) [97]	2001–2007	Female diabetics	100,507	20,571	20.50%	2436	2.40%	11.80%
Stroke	Jackson (2012) [97]	2001–2007	Female non-diabetics	2,630,482			23,632	0.90%	
Stroke	Shestakova (2016) [29]	2015	T2DM adults	3,060,516	66,093	2.20%	8204	0.30%	12.40%
	All CBV deaths		Patients with T2DM	327,168^a^	108,697^a^	3.30%	12,582^a^	1.50%	11.0%^a^

*CAD* coronary artery disease (variously reported as coronary heart disease or ischemic heart disease), *CBV* cerebrovascular disease, *CHF* congestive heart failure (also reported simply as heart failure), *CVD* cardiovascular disease, *MI* myocardial infarction, *NR* not reported, *SCD* sudden cardiac death, *T2DM* type 2 diabetes mellitus

^a^Weighted average of rates taken only for patients with T2DM and where complete outcomes were reported; thus, the number does not represent the sum of all of the numbers in the column above it

### CVD among obese vs. non-obese people with T2DM

About half of the patients included in this analysis had obesity. Three-quarters of the included studies reported on patients’ BMI or the percent of patients with obesity. While the definitions and BMI cut-off points of obesity varied across studies, the most commonly used definition of obesity was a BMI ≥ 30 kg/m^2^, which was employed by 16 studies (43% of those providing a definition).

Five papers reported prevalence rates of CVD according to obesity status, and all of them found a positive relationship between obesity and increased prevalence rates of CVD [26, 37–40]. Using logistic regression to control for multiple factors, Bhatti et al. [26] found a positive correlation between obesity and CAD (P = 0.021). Tamba et al. [37] reported positive correlations between obesity and both CAD (r = 0.3, P < 0.001) and stroke (r = 0.5, P < 0.001). Boonman-de Winter et al. [38] quantified the relationship between BMI and heart failure. The prevalence rate of heart failure was 38.7% (95% CI 31.2–46.1%) in patients with a BMI ≥ 30 kg/m^2^ and 23.4% (95% CI 19.4–27.5%) in those with a BMI < 30 kg/m^2^, which represents a 65% increase due to obesity.

Two studies explored the relationship between increasing BMI and risk of CVD [39, 40]. According to Wentworth et al. [39], for CAD in both males and females, the prevalence rate of CAD increased with each successive increase in BMI, with a five-fold increase between the lowest and highest categories [< 25 (normal), 25–30 kg/m^2^ (overweight), 30–35 kg/m^2^ (mild obesity), 35–40 kg/m^2^ (moderate obesity) and > 40 kg/m^2^ (severe obesity)]. The difference was that prevalence rates in males were about double those for females in every BMI category. For the outcome stroke/transient ischemic attack (TIA) in males, only the highest category (BMI > 40) had elevated prevalence rates, which were about double those for the lowest category (BMI < 25). For females, prevalence rates of stroke/TIA increased in those who were overweight and had mild or moderate obesity but decreased for those with severe obesity. Finally, Glogner et al. [40] had quite different results. They reported a steady increase in prevalence rates of MI from 6.86% in those with a BMI < 20–9.33% in patients who were overweight (BMI 25–30), a 36% increase. However, MI prevalence rates declined thereafter with each increasing category of obesity. The highest category (BMI ≥ 40) had a prevalence rate of 5.01%, which was 27% lower than those in the lowest category (BMI < 20). Thus, patterns vary quite widely, and studies often examined different outcomes.

### Risk of bias in included studies

In the assessment of risk for bias, the studies addressed 80% of the STROBE checklist items (i.e., research design and data presentation), on average. The mean was 80 ± 12%, and the median was 81%, with a range of 54–100%. The two items that were addressed by 100% of the articles were reporting of outcome data and reporting of outcomes. The two items addressed the least were the statement of funding (56%) and indicating the study design with a commonly used term in the title or abstract (60%).

## Discussion

In this systematic review of 4,549,481 persons with T2DM, we estimated the overall prevalence of CVD at 32.2%. The most frequent type of CVD reported was CAD (21.2%) and lowest was stroke (7.6%). Males had higher rates of prevalent disease than females. CVD was responsible for 50.3% of all deaths in T2DM patients over the period of the review. Along with diabetes, cardiovascular disease is associated with several risk factors, obesity, and age. We, therefore, evaluated the association between age and obesity among patients with CVD and T2DM in the selected articles.

### Age as a risk factor for CVD

Age is a well-known risk factor for CVD. Out of the 57 articles, thirteen (25%) reported on the relationship between age and CVD and the results were quite mixed. Nine studies identified a significant relationship between age and CVD [30, 38, 41–47], but only two presented results across multiple age categories [38, 42]. Alonso-Moran [42] found that the odds ratio for IHD, stroke, heart failure and MI all increased sequentially with each increase in 5-year age category as compared with the age group 35–39 used as a reference. All of these individual outcomes achieved statistical significance (P < 0.001). Boonman-de Winter et al. [38] similarly reported a sequential increase in prevalence rates of heart failure for all patients in 5-year age categories from 60 to 64 to > 80 years of age. Other authors reported that older patients had higher prevalence rates than younger patients, but provided few details on age categories [30, 41, 43, 45, 46]. On the other hand, four studies reported no differences between age categories [26, 34, 48, 49]. Three other studies used age as a covariate in a logistic regression with no further details [28, 50, 51]. Therefore, few studies have quantified the effect of age on CVD prevalence rates among people with T2DM.

### Obesity as a risk factor for CVD

Obesity has long been established as an independent risk factor for CVD [7, 52], and is associated with CAD [53, 54], atherosclerosis [51], and cardiac death [55, 56]. Furthermore, it has been shown that overweight and obesity are highly prevalent in T2DM patients with high CV risk and that BMI and waist circumference are related to major cardiometabolic risk factors such as hypertension and elevated low-density lipoprotein cholesterol (LDL-C) [57].

Obesity is usually defined by body mass index (BMI, calculated as body weight in kg divided by the square of height in meters), with the World Health Organization (WHO) classifying adults with a BMI 30 kg/m^2^ as obese [58]. However, BMI as a measure to stratify patients with obesity has limitations and does not account for the wide variation in body fat distribution nor the quality of fat, and may not account for associated health risk in different individuals and populations [58]. This has been shown to be true for South Asian populations [59]. In a study from Raji et al. [60] noted that compared with Caucasians, Asian Indians had significantly greater total abdominal and visceral fat matched with Caucasians of the same age, gender, and BMI, meaning that this population has an increased CVD risk. Besides, there is a weaker association between increasing BMI and T2DM in Asian populations compared with Caucasians due to the risk for T2DM begins increasing at comparatively normal BMI in Asian populations [61].

Seven of the included studies evaluated the relationship between obesity and/or BMI and CVD risk. Five of the studies included in this review identified a positive relationship between obesity and increased prevalence rates of CVD [26, 37–40]. One of these studies [26] used lower BMI cut-off points to account for Asian populations in accordance with WHO recommendations on BMI for Asian populations [62] and evaluated abdominal adiposity with waist circumference measurements to determine the prevalence of obesity. Overall, the studies found a positive relationship between increasing BMI and CVD; except in one study [39], where women with severe obesity had a reduced prevalence of stroke. While the authors do not explain the reduced prevalence of stroke/TIA, it may be explained by differences in vascular risk markers in men, such as pre-existing ischemic heart disease, age, and smoking [63]. Furthermore, the presence of gonadal steroids, most notably estrogen, may lend a protective effect against stroke/TIA in women and it has been shown that adiposity is associated with increased levels of estrogen [64].

Although obesity is identified as a risk factor for CVD, it is associated with a paradox in that mortality is lower in patients who are overweight or obese than in those whose BMI is normal or underweight [65]. Lee et al. [66] reported that obesity provided a survival benefit to patients with heart failure who did not have comorbid diabetes, but not in patients who did have concomitant diabetes. In contrast, a group led by Abi Khalil [67] examined a cohort of 2492 T2DM patients in seven countries in the Middle East, Gulf region, with acute heart failure. They reported that BMI was inversely correlated with the risk of mortality, with severe obesity associated with less mortality risk.

It is clear that the relationship between obesity and the risk of CVD and CVD-related deaths requires further exploration to identify these mechanisms and relationships.

### CVD-related mortality in T2DM

In persons with T2DM, CVD is responsible for at least half of the mortality, as previously mentioned. Among the specific diseases within that term, CAD was most lethal, followed by stroke. Similar results have been demonstrated with other models. In an incidence-based study, Straka et al. [68] followed 29,863 patients (5501 with T2DM and 24,362 without T2DM) over a 1-year period. Four of the incident cardiovascular outcomes they reported were significantly higher in those with T2DM. Patients with T2DM had 10% greater risk of CAD, 53% of MI, 58% of stroke, and 112% increased risk of heart failure. Therefore, T2DM is a substantial risk factor for CVD and its consequences.

### CVD prevalence rates across regions and countries

As this was a global review, studies from across the world were included. Given the variation in which diabetes and its macrovascular complications are treated and managed across countries and income levels, it is relevant to look at prevalence rates across regions and countries. However, almost half (46.0%) of the research was produced in Europe, and very little information was obtained from the less developed regions of the world such as Africa, Latin America, and the Asian subcontinent.

As shown in Table 2, the regions with the highest prevalence of overall CVD were North America and Caribbean (46.0%; N = 4,327,503), Southeast Asia (42.5%, N = 537) and Western Pacific (including China) (33.6%; N = 44,062). Southeast Asia stands out with a higher prevalence of CAD (29.4%) compared with other regions. The prevalence of CAD in this region is driven by one study from India [69], which specifically investigated the pattern of CAD in 134 symptomatic T2DM patients in India. However, epidemiological studies on people of South Asian origin have shown an increased likelihood of developing CAD that is up to two times higher than in Caucasians [70]. The higher risk is due to both pathophysiological and life course-related risk factors [70].

**Fig. 2:**
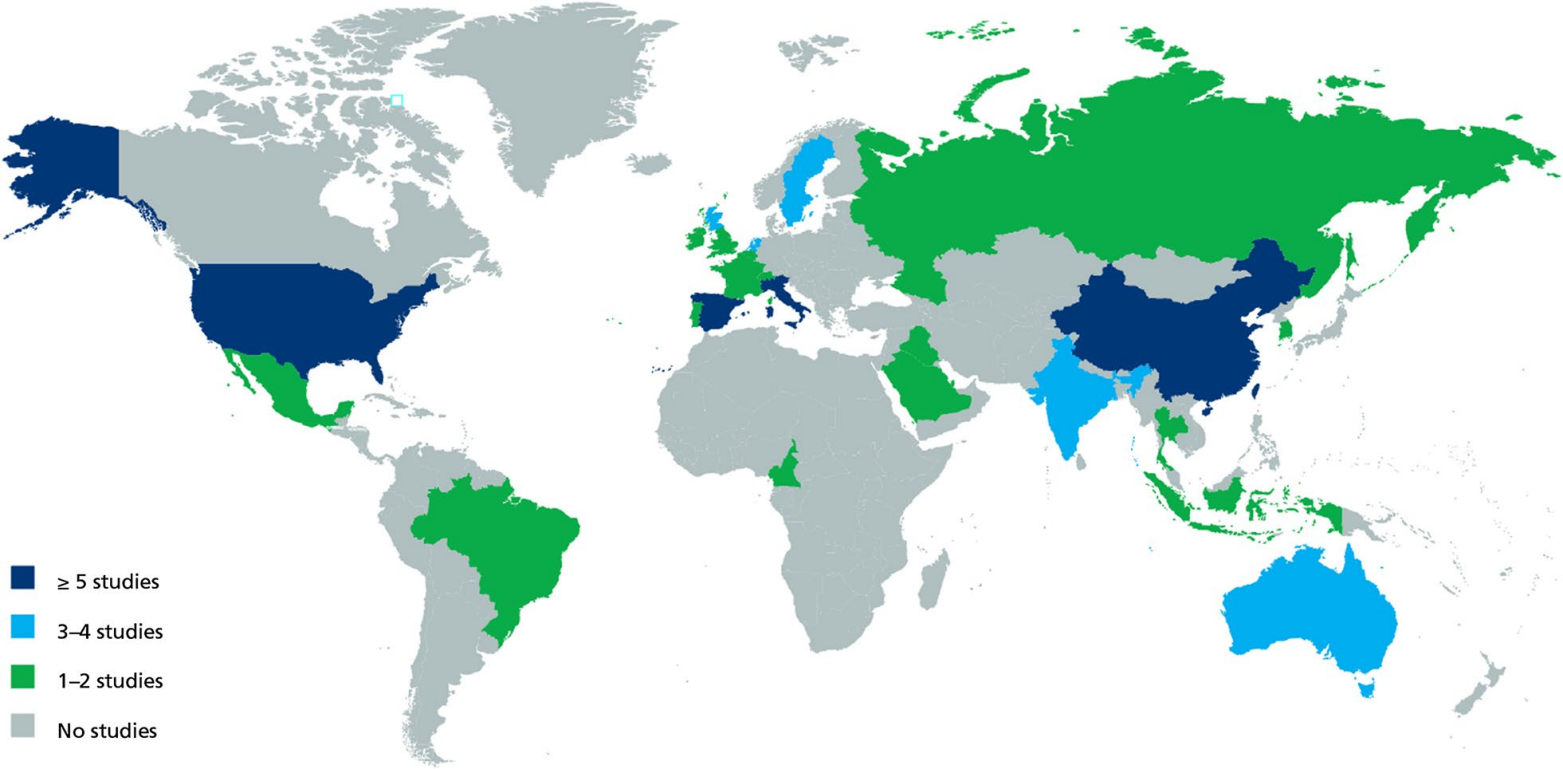
Distribution of studies across countries and regions. The figure illustrates the global distribution of studies across countries and regions

The summaries across countries and regions provide an overview of the geographic spread of research but should be interpreted with caution given the limited number of studies for some of the regions and countries. Figure 2 illustrates the distribution of studies across regions and countries and clearly shows that few studies exist for several regions. For example, only one study from one country in the African region was identified and therefore should not be seen to represent findings for the region’s entire T2DM population.

Treatment of both T2DM and CVD vary greatly between and within countries, and although much of the CVD risk in T2DM can be associated with the long-term complications of T2DM, there has been growing interest to determine whether certain antidiabetic drugs influence this risk. For example, sulfonylureas which are the second most commonly used antidiabetic drug after metformin, have been shown to be associated with an increased risk of cardiovascular events and mortality [71]. Newer antidiabetic drugs have been shown to lower the risk of CVD in T2DM patients [17–20]; however, these drugs are often intended to be used as second- to third-line treatments and many years may pass before patients can benefit.

### Temporal trends in CVD risk assessment and management in T2DM

Encouragingly, CVD mortality is declining in high-income countries among the general population due to reductions in cardiovascular risk factors as well as to recent advances in prevention, treatment, and management [72]. This trend has also been observed in people with T2DM in some countries. Jung et al. [73] estimated trends in CVD in people with and without T2DM in South Korea using data from the national health insurance system. The results show a significant reduction in CVD risk among people with T2DM brought on by improvements in the care and management of patients. However, in many developing countries where the burden of T2DM is rapidly rising and lifestyle patterns changing an increase in CV risk factors among people with T2DM can be expected [3]. A study from China analyzed the relationship between lifestyle behaviors and multiple CV risk factors in 25,454 people with T2DM [74]. The researchers found that unhealthy lifestyles were common, especially among those who are non-elderly, and above-college educated. Furthermore, it was found that an unhealthy lifestyle was associated with poor blood, blood lipid, and blood pressure control. Decreasing the impact of T2DM and CVD in developing countries will require interventions aimed at changing risky lifestyle behaviors.

Screening people with T2DM for CV risk is an important strategy for reducing mortality and CVD events. A study from Denmark [75] found that a single round of diabetes screening and cardiovascular risk assessment in middle-aged adults in general practice was associated with a significant reduction in risk of all-cause mortality and CVD events in people with T2DM. The same researchers found that population-based stepwise screening for T2DM and CVD among all middle-aged adults was not associated with a reduction in mortality or CV events. Therefore, the benefits of population-based screening are limited in this context [76]. Kesall et al. [77] found that targeting specific occupational and industry groups with health checks could help identify individuals at high risk of both T2DM and CVD. In a study of 500,000 members of the Australian working population, they found that high T2DM and CVD risk was increased significantly in many occupational groups and industries.

Recent research points to an increasingly better understanding of the markers for identifying high CVD risk in people with T2DM. Li et al. [78] found that the combined application of carotid and lower extremity ultrasonography may be helpful to identify patients with T2DM who have a higher CVD risk. In a study of 2830 hospitalized patients with T2DM, they found that the concomitant presence of carotid and lower extremity atherosclerosis further increases the risk of CVD in patients with T2DM, compared with those who had either carotid or lower limb atherosclerosis and those without atherosclerosis. A study by Mohammedi et al. [79] found that major peripheral arterial disease (PAD) presenting as lower-extremity ulceration or amputation and peripheral revascularization is associated with increased risk of death and CV events in people with T2DM. The researchers conclude that screening for PAD along with active management are crucial for prevention of CVD in people with T2DM. In addition, coronary artery calcium (CAC) assessments have been found to significantly improve the risk classification for CHD and atherosclerotic CVD events in people with T2DM—regardless of the duration of diabetes [80]. Thus, a CAC assessment can be a useful tool for classifying people with T2DM into lower- or higher-risk groups for long-term CVD risk.

Lipid profile has long been considered among the most important risk factors for CVD in T2DM, and several trials have confirmed that lowering low-density lipoprotein cholesterol (LDL-C) via statins in T2DM was effective in reducing the risk of CVD [81, 82]. It is also well known that statins also have a triglyceride-lowering effect [83]. In a cross-sectional study of 223,612 patients with T2DM in China, researchers found that although lower triglyceride was associated with reduced CVD risk in the short-term, it was associated with increased risk in the long-term [84]. This paradox could mean that low triglyceride is not necessarily associated with good clinical outcomes in all people with T2DM and that there are subgroup associations with CVD in patients with different durations of T2DM. Furthermore, Clua-Espuny et al. [85] suggest that the relative importance of risk factors wanes in complex chronic patients with T2DM with advancing age. In a cohort study of almost 3500 complex chronic patients above the age of 80 of whom 53% had diabetes and a high prevalence of associated classical risk factors, the researchers found that all-cause mortality was more affected by aging factors than by specific complications of diabetes. The authors make the recommendation that, for these patients, the care strategy may need to be redefined and adapted to comorbidities and functional autonomy rather than being focused on treatment outcomes.

## Limitations

As with all literature reviews, we were limited by the availability of the literature and the validity and quality of the articles. Some of the results appeared only in abstract form, and many were not subsequently published as full articles within the time horizon of this review. Abstracts had space limitations, restricting the amount of information they could present. As well, we noted that there was often incomplete reporting or selective reporting of specific outcomes of interest.

Furthermore, the findings of this literature review are limited to a select patient population. Specifically, in this research, we accepted only data from adults aged 18 or older. Therefore, our results may not apply to children or adolescents. As well, we dealt only with T2DM; therefore, outcomes may not apply to T1DM or secondary diabetes such as that associated with hemochromatosis or pancreatitis.

This study was also challenged by the fact that CVD and its associated conditions are described differently across the literature. For example, CHD was used interchangeably with CAD or ischemic heart disease. We made every effort to standardize definitions and to group like with like. Furthermore, the types of CVD conditions evaluated varied across articles. Some articles focused on a single outcome, whereas others focused on several outcomes. As a result, the calculated prevalence rates may represent underestimates, as not all studies reported all outcomes.

The types of studies included would have also impacted the overall results of this study. First, we analyzed only prevalence studies; incidence studies would have different results due to their different perspective. Second, the studies varied both in the method of data collection (e.g., national databases versus clinic records) and the length of time over which they collected data. It is plausible that time-period over which studies were conducted could have impacted the observed prevalence rate of CVD. For example, health status, lifestyle, and treatments have varied over time, which could impact the prevalence rates in the studies using older data.

Overall, it is possible that the prevalence estimates for CVD presented in this article overestimate the prevalence of CVD among patients with T2DM. First, studies in the medical literature tend to include a sicker population compared to the general T2DM population; therefore, due to self-selection bias, the sample may not be representative of the broader T2DM population and thus lead to an overestimate of the prevalence of CVD. Second, some of the studies included T2DM patients with an existing CVD diagnosis; therefore, the overall estimate of CVD within these studies could be higher compared to the broader T2DM population.

Finally, only 25 countries were represented in this analysis. Noticeably absent were such countries as Germany, Canada, and Denmark, which all have excellent electronic health data, yet no research studies have been published from them. Very little has appeared from Africa, the Asian subcontinent or Latin America. More studies from these areas would be welcome. While the scope of this study was to evaluate evidence from peer-reviewed literature, an alternative approach to estimating the prevalence of CVD among patients with T2DM could be to analyze data within existing registries.

## Conclusions

This is the first systematic review to synthesize global prevalence rates of CVD, including stroke, MI, angina, heart failure, atherosclerosis and CAD among people with T2DM. The results show that CVD is a major cause of comorbidity and death among patients with T2DM with CAD having the highest prevalence. There is a paucity of research studies investigating both the prevalence of CVD and risk factors such as obesity among people with T2DM. Given the large burden that CVD exerts on healthcare systems, patients and families around the world, more evidence is needed, ideally in the form of registry studies, to more accurately quantify the global prevalence of CVD among people with T2DM.
